# The Effect of Spectral Quality on Daily Patterns of Gas Exchange, Biomass Gain, and Water-Use-Efficiency in Tomatoes and Lisianthus: An Assessment of Whole Plant Measurements

**DOI:** 10.3389/fpls.2017.01076

**Published:** 2017-06-20

**Authors:** Jason Lanoue, Evangelos D. Leonardos, Xiao Ma, Bernard Grodzinski

**Affiliations:** Department of Plant Agriculture, University of GuelphGuelph, ON, Canada

**Keywords:** water-use-efficiency, light-emitting diodes (LED), tomatoes, lisianthus, gas exchange, transpiration, whole plant, greenhouse

## Abstract

Advancements in light-emitting diode (LED) technology have made them a viable alternative to current lighting systems for both sole and supplemental lighting requirements. Understanding how wavelength specific LED lighting can affect plants is thus an area of great interest. Much research is available on the wavelength specific responses of leaves from multiple crops when exposed to long-term wavelength specific lighting. However, leaf measurements do not always extrapolate linearly to the complexities which are found within a whole plant canopy, namely mutual shading and leaves of different ages. Taken together, both tomato (*Solanum lycopersicum*) leaves under short-term illumination and lisianthus (*Eustoma grandiflorum*) and tomato whole plant diurnal patterns of plants acclimated to specific lighting indicate wavelength specific responses of both H_2_O and CO_2_ gas exchanges involved in the major growth parameters of a plant. Tomato leaves grown under a white light source indicated an increase in transpiration rate and internal CO_2_ concentration and a subsequent decrease in water-use-efficiency (WUE) when exposed to a blue LED light source compared to a green LED light source. Interestingly, the maximum photosynthetic rate was observed to be similar. Using plants grown under wavelength specific supplemental lighting in a greenhouse, a decrease in whole plant WUE was seen in both crops under both red-blue (RB) and red-white (RW) LEDs when compared to a high pressure sodium (HPS) light. Whole plant WUE was decreased by 31% under the RB LED treatment for both crops compared to the HPS treatment. Tomato whole plant WUE was decreased by 25% and lisianthus whole plant WUE was decreased by 15% when compared to the HPS treatment when grown under RW LED. The understanding of the effects of wavelength specific lighting on both leaf and whole plant gas exchange has significant implications on basic academic research as well as commercial greenhouse production.

## Introduction

During the past few decades, technical advancements have made light emitting diodes (LEDs) a viable, new source for both sole and supplemental lighting systems in controlled environments used for basic research purposes, as well as commercial production of fresh produce in greenhouses (Nakamura et al., 1994; Tepperman et al., 2004). Much of what we know about photosynthesis driven by LEDs comes from studies of leaf gas exchange (Goins et al., 1997; Kim et al., 2004a; Hogewoning et al., 2010; Liu et al., 2012).

Leaf measurements have been used as a proxy to understand how the more complex whole plant canopy functions under different environment conditions (Liu et al., 2011a). However, it is well known that leaf metabolism is not a perfect proxy for whole plant metabolism and growth due to metabolic activity of organs/tissues other than leaves, mutual shading, studying leaves of different ages, and differences in canopy architecture including leaf orientation (Davis and McCree, 1978; De Vries, 1982; Dutton et al., 1988). Taken together, the complexities of whole plant photosynthetic and respiratory gas exchange plus canopy architecture do not allow for simple extrapolation of leaf gas exchange kinetics to whole plant metabolic measurements. Whole plant measurements provide the additional data regarding the nature of the plant canopy to be considered. It, therefore, is these gas exchange data collectively that provide a powerful, non-destructive, means of estimating daily growth patterns of the entire plant when subjected to different light, CO_2_, and temperature conditions (Dutton et al., 1988; Leonardos et al., 2014).

It is well known that plant biomass production increases with the amount of light given to the plant (Evans and Hughes, 1961). Recent improvements in LED technology has led to lower production costs and manufacturing of lighting systems for plant growth (Nakamura et al., 1994; Nelson and Bugbee, 2014; Singh et al., 2015). While conventional high pressure sodium (HPS) lighting have some advantages over LEDs, several characteristics of LEDs allow for the potential optimization of lighting treatments including: (i) ability to provide wavelength specific lighting, (ii) a cool light emitting face, and (iii) potential use as inner canopy lighting (Nakamura et al., 1994; Nelson and Bugbee, 2014). An important aspect of the commercial application of LEDs as supplementary lighting is how plant production can be optimized while the greenhouses are also receiving natural solar radiation at different times of the year (Runkle and Heins, 2001; Kim et al., 2004b; Gomez and Mitchell, 2015; Rabara et al., 2017).

One of the major advantages of LED lighting is the ability to administer wavelength specific lighting. Since the early 1940s, it has been known that the main photosynthetic pigments of a leaf, chlorophyll *a* and *b*, preferentially absorb certain wavelengths of light (red and blue; Mackinney, 1941). It has been determined that not only are different wavelengths from LED lights absorbed differently, but they are also able to create different plant morphological traits, alter leaf anatomy, cause different stomatal responses, alter flowering time, and even alter gene expression in a variety of plant species (Runkle and Heins, 2001; Tepperman et al., 2004; Liu et al., 2011a,b; Gomez and Mitchell, 2015; Snowden et al., 2016; Rabara et al., 2017).

Advancements in genetic engineering and breeding places an entirely new emphasis on plant phenotyping approaches that should consider and differentiate gas exchanges of the leaf (primary photosynthetic organ) and those of the more complex plant canopy (whole plant organism; Leonardos and Grodzinski, 2016). To our knowledge, current plant literature lacks studies that indicate how the new LED lighting systems might be affecting photosynthetic, respiratory and water exchanges at the whole plant level over the course of a diurnal period, not merely during short-term measurements.

Currently, there is little information dealing with the inter-relationships among transpiration, water use, and growth patterns of plants that are being grown under LEDs. What little data on water-use-efficiency (WUE) and growth patterns that are available have come from interpretation of gas exchange at the leaf level. In this study, we provide data showing relationships between light quality produced by different LED fixtures on the diurnal whole plant net carbon exchange rate (NCER), transpiration rate, and WUE of a major vegetable crop, tomato (*Solanum lycopersicum*) and an ornamental cut flower, lisianthus (*Eustoma grandiflorum*), both requiring supplemental lighting when these crops are produced in controlled greenhouse environments during winter periods.

## Materials and methods

### Plant material and growth conditions

Seeds of *S. lycopersicum* cv. “Bonny Best” (BB) were obtained from William Dam Seeds (Dundas, ON, Canada). Seeds were sown in 60 cavity potting trays in Sungro professional growing mix #1 (Soba Beach, AB, Canada) between December 2015 and March 2016 and placed in a greenhouse misting bed. Plantlets were then transferred to larger 1L pots containing Sungro growing mix #1 and these were placed in our research greenhouse at the University of Guelph (43° 31′ 40.0584″ N, 80° 13′ 38.4996″ W).

*E. grandiflorum* cv. “Flare” (Lisianthus) rooted plantlets were obtained from John Slamans Greenhouse Ltd., (Burford, ON, Canada). Lisianthus plants were also raised in pots in the same greenhouse in a similar manor as the tomatoes.

**Figure 1:**
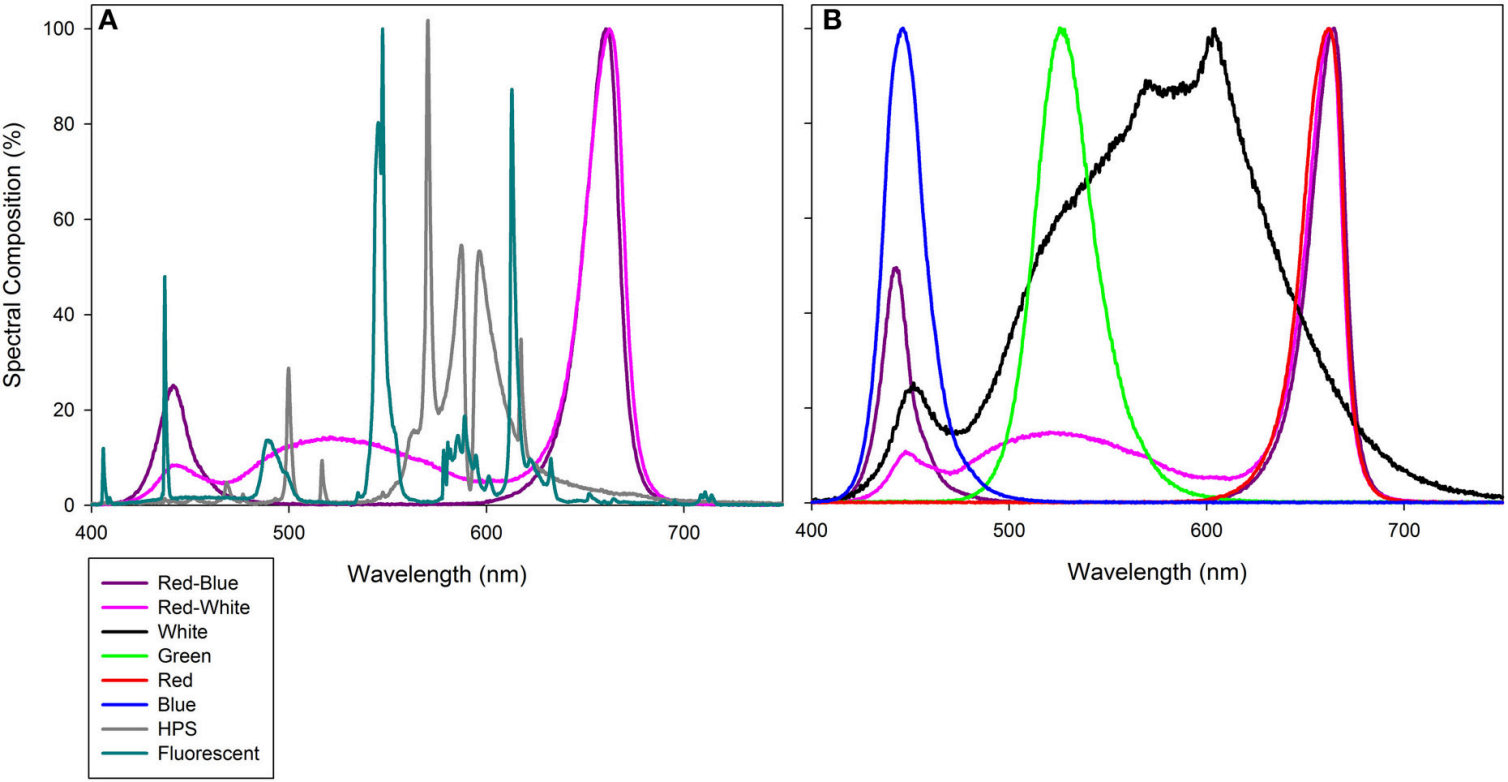
Photosynthetically active radiation (PAR) spectrum of compact fluorescence growth light within the Bio-chambers for plants involved in leaf studies **(A)**, as well as HPS, RB LED, and RW LED from Philips Lighting Company and LSGC, respectively which were used during both greenhouse grow periods and during whole plant analysis. **(B)** provides the PAR spectrum of PAR 38 LED floodlights from LSGC. All light spectra were determined using a spectroradiometer (Flame Spectrometer, Ocean Optics, Dunedin, FL, USA). Spectral composition (%) of each light can be found in Supplementary Table 1.

Tomato and lisianthus plants were placed under 4 different light treatments, each of which was replicated 3 times in a randomized block design. Light treatments included an ambient (natural light/control), and three supplemental light treatments each providing 100 ± 25 μmol m^−2^ s^−1^ of supplemental photosynthetically active radiation (PAR) as determined by a Li-COR quantum sensor (Li-190SA, Li-COR Inc. Lincoln, NE, USA) at pot level at the beginning of the experiment from HPS lights (Agrolite XT; Phillips Lighting, Markham, ON, Canada), red-blue LEDs (LsPro® VividGro® V1 Grow Fixture; Lighting Science Group Company (LSGC) Warwick, RI, USA), and red-white LEDs (LSGC; Figure 1A). Supplementary light was provided for 16 h daily from 6:00 a.m. to 10:00 p.m. Shade curtains were utilized when solar radiation exceeded 500 μmol m^−2^ s^−1^ of PAR. The temperature was maintained at 20°C during the day and night period, relative humidity (RH) was maintained around 55% and plants were watered with fertilizer (20-8-20, Micronutrients; Boron (B) = 200 ppm, Copper (Cu) = 500 ppm, Iron (Fe) = 1000 ppm, Manganese (Mn) = 500 ppm, Molybdenum (Mo) = 150 ppm, Zinc (Zn) = 500 ppm, Magnesium (Mg) = 1500 ppm; pH = 6, EC = 2.3 mS/cm) as needed. Plants grown during this low (natural) light, winter period were used for both whole plant gas exchange studies and destructive biomass analysis.

In addition to growing tomato plants in the greenhouse, populations of BB tomatoes were also grown from seeds in a growth chamber (GC-20 Bigfoot series, Biochambers, Winnipeg, MB, Canada). Temperature was set to 22/18°C (d/n) with a 16/8 h photoperiod. Plants in these growth cabinets were illuminated with 300 ± 50 μmol m^−2^ s^−1^ of PAR as determined by a Li-COR quantum sensor at canopy level supplied by compact fluorescence lights (CFL; Sylvania Pentron 841 HO Ecologic, Wilmington, MA, USA; Figure 1A). The RH was maintained at 60 ± 10% and plants were watered with fertilizer (24-8-16; Miracle Gro™, Micronutrients; Boron (B) = 200 ppm, Copper (Cu) = 700 ppm, Iron (Fe) = 1500 ppm, Manganese (Mn) = 500 ppm, Molybdenum (Mo) = 5 ppm, Zinc (Zn) = 600 ppm; pH = 5.8, EC = 2.6 mS/cm; Marysville, OH, USA) as needed. Plants from these growth cabinets were used for leaf gas exchange measurements.

### Growth analyses of greenhouse grown crops

#### Whole plant gas exchange and diurnal growth patterns

Our current whole plant gas exchange system which is comprised of six polycarbonate/glass chambers is similar to that described previously (Dutton et al., 1988; Leonardos et al., 2003). Gas exchange measurements were made by sampling each chamber for 90s, cycling through all chambers every 9 min throughout the day and night periods over a 36–48 h period (one run). Two chambers were illuminated with the same HPS lights, two chambers were illuminated with the same red-blue LED luminaries, and the final two chambers were illuminated with the same red-white LED luminaries as above. Light treatments were rotated between the chambers every two runs to remove chamber bias and all chambers were wrapped in aluminum foil to ensure light treatments did not enter an adjacent chamber (Supplementary Figure 1).

For analysis of greenhouse grown tomatoes, experiments started on April 7th, 2016 and continued through April 25th, 2016 [36–54 days after sowing (DAS)]. Plants were placed in the chambers the day before around 3:00pm and measurements used for the calculations were taken from the following day/night period. Lights were set to 500 ± 10 μmol m^−2^ s^−1^ for each light treatment as determined by a Li-COR quantum sensor at the top of the plant and set to a 16/8 h photoperiod. Plants which were grown under supplemental light in the greenhouse (acclimated plants) were analyzed under the same light treatment (i.e., grown under red-blue 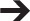 analyzed under red-blue) and plants which were grown under ambient lighting (non-acclimated plants) were analyzed under all supplementary light treatments. Temperature inside the chambers was set to 22/18°C with a RH of 55 ± 5% and 400 μL L^−1^ CO_2_. The following morning after data collection, plants were removed from the chambers and leaf area was measured using a leaf area meter (Li-COR 3100, Li-COR Inc. Lincoln, NE, USA) and all NCER values were normalized on a leaf basis. Three runs from each block were done amounting in 9 replicates per light treatment.

Analysis involving lisianthus began on March 3rd, 2016 and continued through March 24th, 2016 (120–141 DAS). The same protocol was used as the tomatoes, however conditions were as follows: temperature inside the chambers was set to 22/20°C with the RH set to 50 ± 5% and a CO_2_ concentration of 400 μL L^−1^. Again, plants grown under supplemental light were analyzed under the same light they were grown under and ambient plants were analyzed under all 3 supplementary light treatments.

#### Biomass partitioning (destructive analysis)

Tomato plants (97 DAS) were destructively analyzed for their end biomass production under their lighting treatments. Two plants from each block (6 plants per light treatment) had their leaf area measured with a leaf area meter and all leaf material (leaves, stems, roots, and flowers) were dried in an oven for 48 h at 70°C, allowed to cool for an hour then weighed. An identical protocol was used to determine biomass partitioning within lisianthus (189 DAS). Six plants were taken from each ambient block (18 plants in total) and 4 plants were taken from blocks with supplemental light (12 plants per supplementary light treatment).

### Leaf gas exchange under monochromatic and multicolour LEDs

Leaf gas exchange begun on chamber grown tomato plants 31–35 DAS. The fifth, fully expanded leaf was placed in the chamber of a Li-COR 6400 (Li-COR Inc. Lincoln, NE, USA) which was fitted with a clear top chamber. The leaf temperature within the chamber was held at 22°C with a relative humidity of 50–60% and a CO_2_ level of 400 μL L^−1^. Lights used to generate the leaf gas exchange curves were specially designed LED flood lights (PAR 38, LSGC). The effect of spectral quality on gas exchange was determined using diodes producing the following peak wavelengths: red (R; 660 nm), blue (B; 440 nm), orange (O; 595 nm), green (G; 500 nm), white (W), red-blue (RB), or red-white (RW; Figure 1B). Three different leaves, each from a different plant, were used for each light treatment. Light curves began at a high light intensity and decreased incrementally which follows the procedure from Evans and Santiago (2014). At each light level, the photosynthetic rate was allowed to steady, then a 2 min period was averaged to produce photosynthetic, C_*i*_, and transpiration values for that light level. Photosynthetic rates were then plotted against light intensity and a regression line following the equation f = y_*o*_ + a(1−e^(-b*x)^) was applied, where y_*o*_ is the respiration rate at 0 μmol m^−2^ s^−1^ of light, a is the maximum Pn rate (μmol m^−2^ s^−1^), and b is the quantum efficiency. The regression line was applied in SigmaPlot to the data points in order to calculate the parameters presented in **Table 1**.

### Statistical analysis

All statistics were performed used SAS studio 3.5. A one-way ANOVA was performed with a Tukey Kramer adjustment at *p* < 0.05 to determine differences between mean values. Outliers were determined by examining internal studentized residuals and comparing them to the Lund's critical value via a Lund's test (Lund, 1975).

## Results

### Whole plant gas exchanges

**Figure 2:**
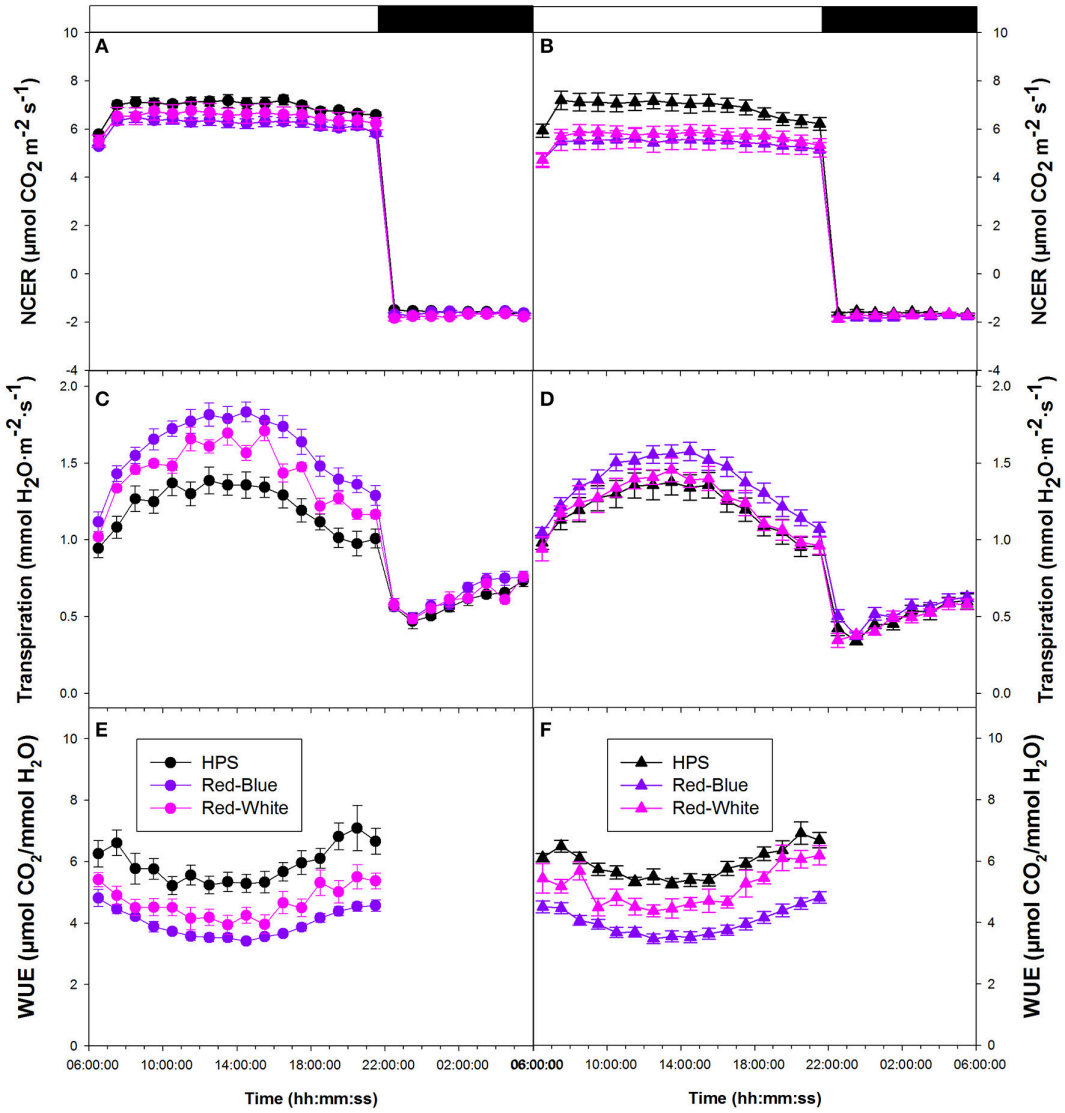
Whole plant diurnal patterns of NCER **(A,B)**, transpiration rate **(C,D)**, and WUE **(E,F)** of lisianthus plants tested under HPS and RB or RW LED lights, but grown in the greenhouse only under ambient light **(A,C,E)** or with supplemental light **(B,D,F)**. Whole plant points represent the hourly mean values ± the standard error of 12 replicates for panels **(A,C,E)** and 6 replicates for panels **(B,D,F)**.

Figure 2A compares primary whole plant NCER of lisianthus that were grown under only the ambient light conditions in the greenhouse, but were then measured using the three different lighting systems (i.e., HPS, RW LED, and RB LED). In comparison, Figure 2B shows whole plant NCER of lisianthus plants that were grown in the greenhouse under each of the three artificial lighting systems and then measured under these lights. Importantly, the data in Figure 2A, showing non-acclimated plants to the artificial lights, and those in Figure 2B, showing acclimated plants, are very similar and show steady photosynthetic rates during the day. Interestingly, plants which were grown under HPS lighting produced an ~18% higher whole plant photosynthetic rate throughout the light period than those plants grown under either LEDs (Figure 2B). The difference in whole plant photosynthetic rate under the HPS vs. the two LEDs was only observed when plants were both grown and tested with the HPS lighting and not in plants grown in ambient conditions but exposed to short-term light treatment (Figures 2A,B). A noticeable drop in day-time NCER rates was seen in the RB and RW LED when under acclimated conditions compared to non-acclimated grow conditions (Figures 2A,B). Regardless of the growth conditions (acclimated or non-acclimated plants), all lisianthus plants had a similar NCER at night indicating that respiration patterns were not affected by the type of day time light treatments in these experiments (Figures 2A,B).

Figures 2C,D show the transpiration rates of the same plants that were described in Figures 2A,B, respectively. Under all light conditions, transpiration rates were not steady during either the light or dark periods (Figures 2C,D). In the first half of the photoperiod, transpiration rates increase to a maximum at 14:00 h and subsequently declined during the afternoon (Figures 2C,D). When the lights were turned off, there was a dramatic decrease in transpiration rate, consistent with stomatal closure in the dark. However, during the night period, all lisianthus plants showed a gradual increase in their transpiration rates under all conditions (Figures 2C,D). Slightly lower transpiration rates were observed from the acclimated plants under RB and RW LED compared to non-acclimated plants (Figures 2C,D). This was not seen in the plants analyzed under HPS plants.

Figures 2E,F show the diurnal patterns of WUE for non-acclimated plants and acclimated plants, respectively, which is a function of both photosynthetic CO_2_ fixation rates (Figures 2A,B) and water loss through transpiration (Figures 2C,D). Under all light treatments, the non-acclimated plants analyzed under the three different artificial light treatments showed a decrease in WUE during the middle of the photoperiod (Figure 2E). This similar pattern was seen in lisianthus plants which had been acclimated to the three artificial light treatments (Figure 2F). Interestingly, in both non-acclimated and acclimated lisianthus plants, the RB LED light treatment was observed to have the lowest WUE among all light treatments tested (Figures 2E,F).

**Figure 3:**
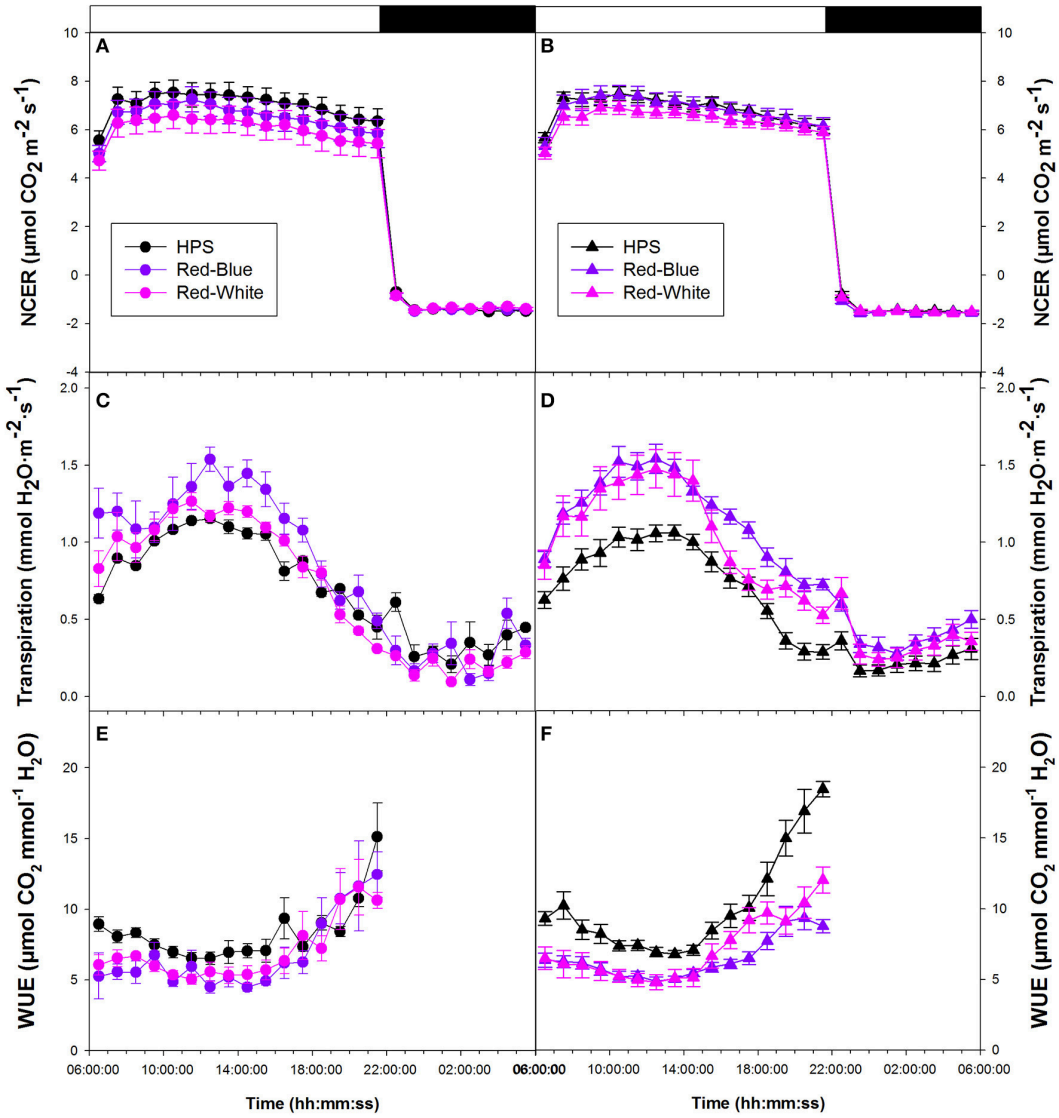
Whole plant diurnal patterns NCER **(A,B)**, transpiration rate **(C,D)**, and WUE **(E,F)** from greenhouse grown tomato plants under ambient light **(A,C,E)** and supplemental light **(B,D,F)**. Whole plant points represent the hourly mean values ± the standard error of 9 replicates for both non-acclimated and acclimated plants.

Figure 3A provides data from tomato plants which were grown in a greenhouse under ambient conditions then subjected to the two LED (RW and RB) and HPS light treatments. Figure 3B shows data which compares plants grown in a greenhouse under supplemental light from either HPS, RW LED, or RB LED. In both experimental conditions, whole plant NCER were seen to be steady throughout both day and night periods (Figures 3A,B). All lighting conditions provided similar rates of both photosynthesis and respiration (Figures 3A,B).

Whole plant transpiration rates seen in Figures 3C,D are from the same non-acclimated and acclimated tomato plants, respectively, as were used in whole plant NCER analysis. Unlike NCER, transpiration rates increased to a maximum around mid-day and declined thereafter in both non-acclimated and acclimated plants (Figures 3C,D). At the maximum transpiration rate, in both non-acclimated and acclimated plants, plants analyzed under the RB LED light treatment showed an increase of ~50% from tomato plants analyzed under HPS lighting. Once the lights were turned off (22:00 h), there was a subtle increase in transpiration rates during the 8 h night period (22:00–06:00 h) under all experimental conditions (Figures 3C,D).

Tomato whole plant WUE of non-acclimated plants showed similar patterns under all light treatments (Figure 3E). Interestingly, there was an increase in WUE from 18:00 to 22:00 h under all light treatments (Figure 3E). Both RW and RB LED light treatments show slightly lower WUE during the morning hours than the HPS light treatment (Figure 3E). Water-use-efficiency of the acclimated plants showed similar patterns to those of non-acclimated tomato plants (Figures 3E,F). At mid-day, WUE values for the RW and RB LED light treatments were 30% below that of the HPS (Figure 3F). Notably, the HPS treatment showed a very dramatic increase in WUE in the late afternoon compared to that measured with either the RW or the RB LED treatment (Figure 3F).

**Figure 4:**
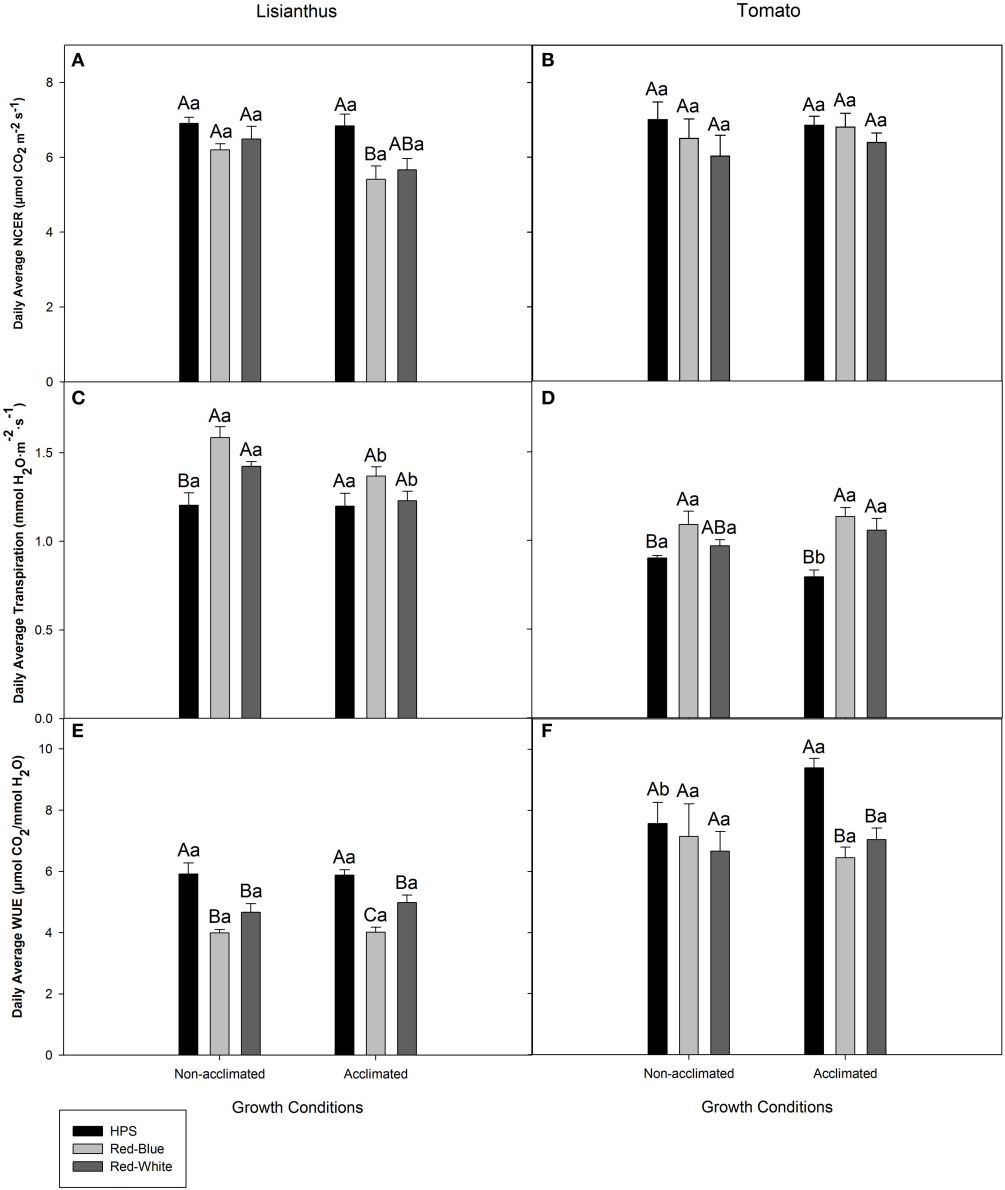
Day-time whole plant average NCER **(A,B)**, transpiration rate **(C,D)**, and WUE **(E,F)** of lisianthus **(A,C,E)** and tomatoes **(B,D,F)** grown in a greenhouse under non-acclimated and acclimated growth conditions. Average lisianthus whole plant data are daily means ± the standard error of 12 replicates for plants grown under acclimated conditions and 6 replicates for lisianthus grown under non-acclimated conditions. Tomato whole plant data are daily means ± the standard error of 9 replicates for both acclimated and non-acclimated growth conditions. Upper case (A–C) represent statistical differences within a panel comparing non-acclimated or acclimated plants within a growth condition, across light treatments. Lower case (a,b) represent statistical differences within a panel between non-acclimated and acclimated plants analyzed under the same light treatment. All statistical comparisons were done using a one-way ANOVA and a Tukey-Kramer adjustment (*p* < 0.05).

Figure 4 shows the average whole plant photosynthesis, transpiration rate, and WUE during the light period for lisianthus and tomato that were derived from data represented in Figures 2, 3, respectively. Whole plant photosynthesis for non-acclimated lisianthus grown under ambient light and then subjected to the three light treatments showed no differences (Figure 4A). However, acclimated lisianthus which were grown under HPS light had a higher average photosynthetic rate compared to the RB LED treatment (Figure 4A). In contrast, the average whole plant photosynthetic rates of the acclimated tomatoes were the same regardless of light treatment (Figure 4B). When comparing non-acclimated vs. acclimated plants of the same species under the same light treatment (i.e., non-acclimated HPS vs. acclimated HPS) no differences in photosynthesis were seen (Figures 4A,B).

The average transpiration rates of non-acclimated lisianthus grown under ambient light then exposed to RW and RB LED were higher than those plants exposed to HPS lighting in the short-term (Figure 4C). No difference was seen in whole plant transpiration rates of acclimated lisianthus plants (Figure 4C). Statistical differences were seen when comparing lisianthus non-acclimated to acclimated plants which were analyzed under RB and RW LED light treatments (Figure 4C).

In non-acclimated tomato plants, the RB LED treatments generated a higher transpiration rate than the HPS light treatment (Figure 4D). However, in acclimated tomato plants, those grown and analyzed under both RW and RB LEDs had higher transpiration rates than did plants in the HPS light treatment (Figure 4D). An increase in transpiration rate from tomato plants acclimated under HPS when compared to non-acclimated plants, analyzed under HPS was observed (Figure 4D).

The highest day-time average WUE of both non-acclimated and acclimated lisianthus plants, was observed when analyzed under the HPS light (Figure 4E). Additionally, in acclimated lisianthus plants grown under the RB LED the WUE was lower than that of the RW LED treatment (Figure 4E). No differences were determined between non-acclimated and acclimated lisianthus under any light treatment (Figure 4E).

In non-acclimated tomato plants, no difference in WUE was observed under the different lights (Figure 4F). Notably, with acclimated tomato plants, a decrease in the average WUE of 31 and 25% was observed under the RB LED and the RW LED, respectively, when compared to the WUE of tomatoes grown and analyzed under the HPS lights (Figure 4F). Also, there was a statistical increase in WUE in HPS acclimated tomatoes when compared to non-acclimated tomatoes which were also analyzed under HPS lights (Figure 4F). There was no difference seen between non-acclimated and acclimated tomatoes analyzed under RB or RW LED (Figure 4F).

### Biomass partitioning

**Figure 5:**
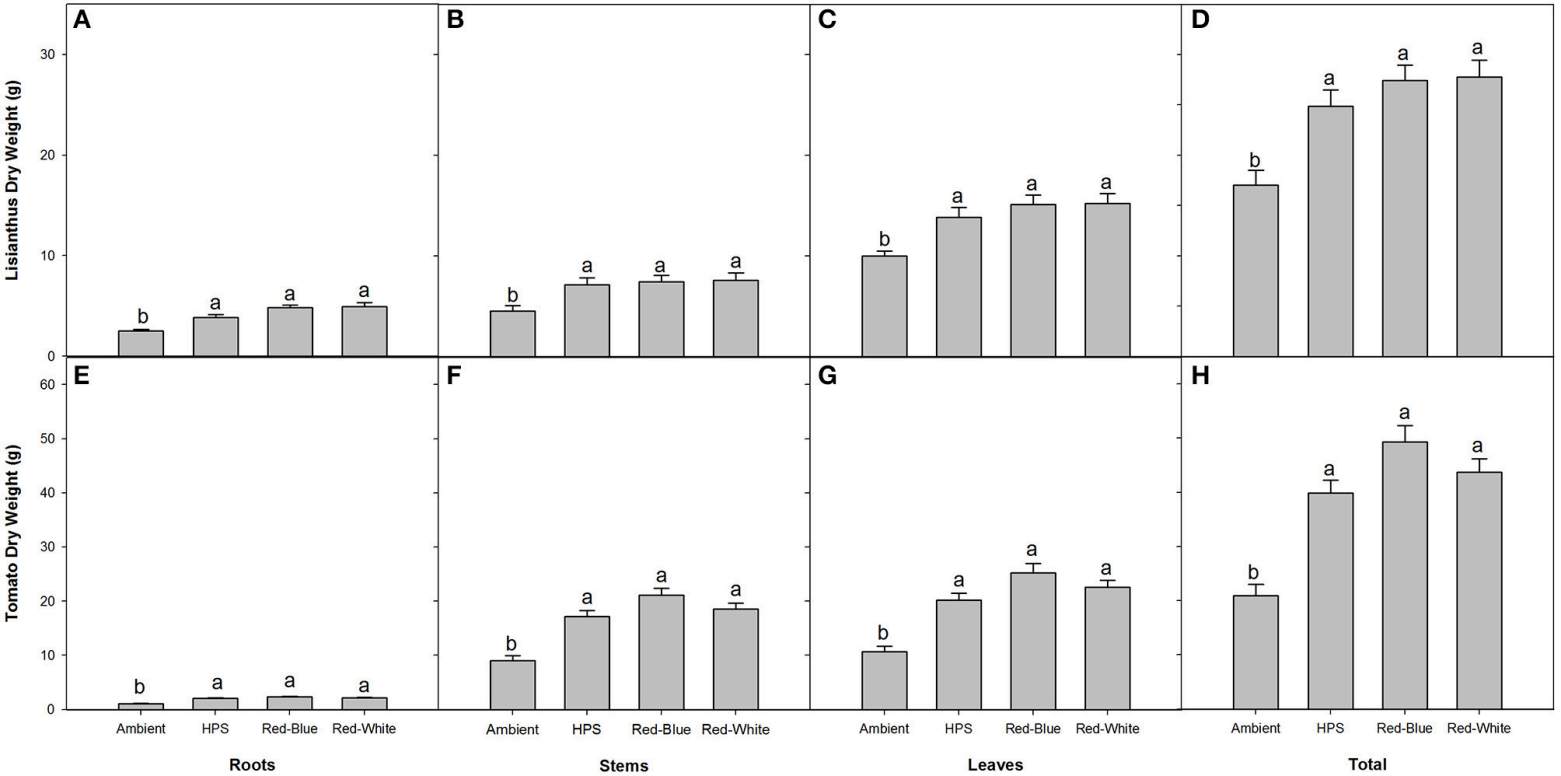
Biomass partitioning of lisianthus **(A–D)** and tomatoes **(E–H)** for plants grown under either ambient, HPS, RB LED, or RW LED light treatments. Lisianthus bars represent the mean of 12 plants for supplemental light grown plants and 18 plants for ambient grown plants ± standard error. Tomato bars represent the mean of 6 replicates ± the standard error for both plants grown under supplemental lighting and ambient lighting. Letters (a,b) represent significant differences within panels as determined by a one-way ANOVA with a Tukey-Kramer adjustment (*p* < 0.05).

In both lisianthus and tomatoes, plants grown under the three supplemental light treatments produced more total biomass than did the plants grown under the ambient light treatment (Figures 5D,H). Also of note, biomass partitioning was similar when plants were grown under the three different light treatments for both crops, respectively, indicating no preferential biomass accumulation between different sink tissues (Figure 5).

### Leaf gas exchange under monochromatic and multicolour LEDs

**Figure 6:**
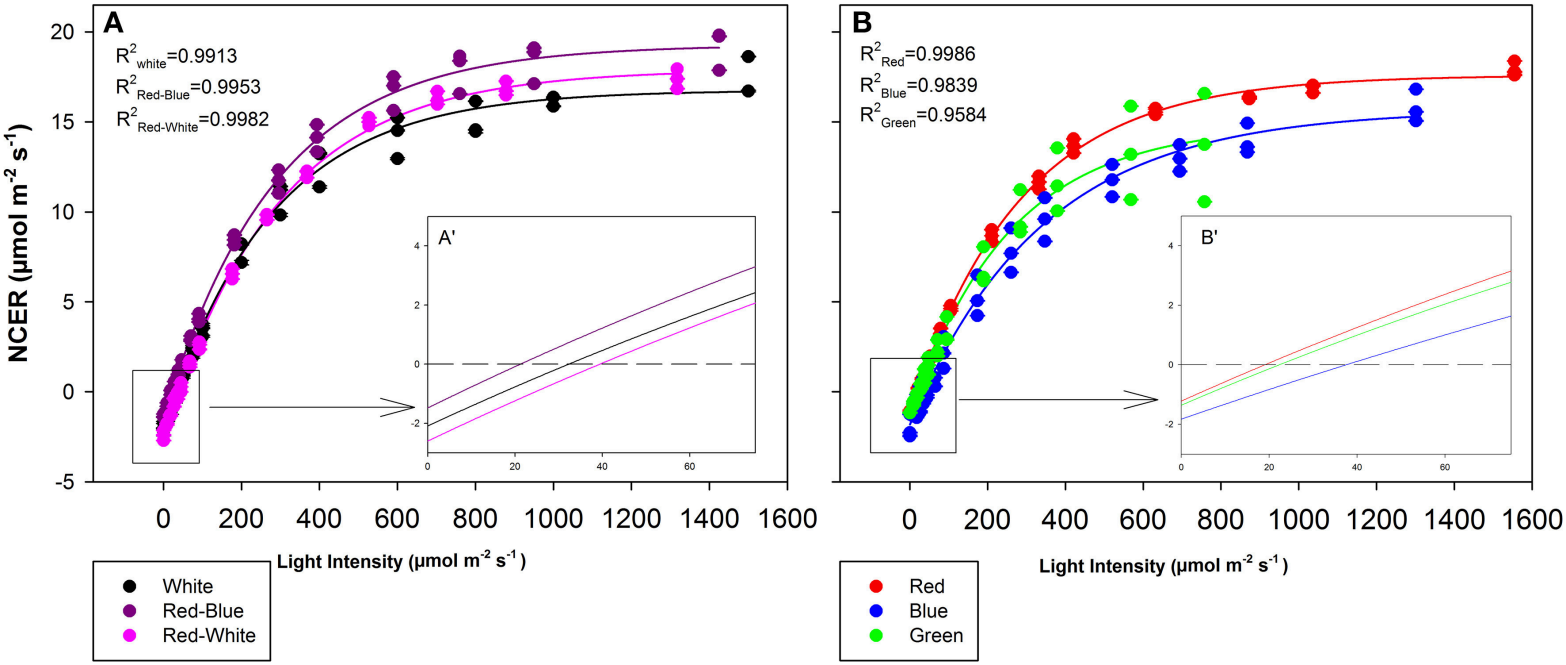
Leaf NCER for mixtures **(A)** and monochromatic **(B)** LEDs of tomato plants which were grown in growth chambers under fluorescent light. Insert A′ and B′ are magnifications of the 0–75 μmol m^−2^ s^−1^ PAR region of panels **(A,B)**, respectively for better interpretation by the reader. Leaf NCER values of 3 replicate leaves shown with a regression lines fit to f = y_o_ + a(1−e^(−b*x)^) for each light treatment.

**Figure 7:**
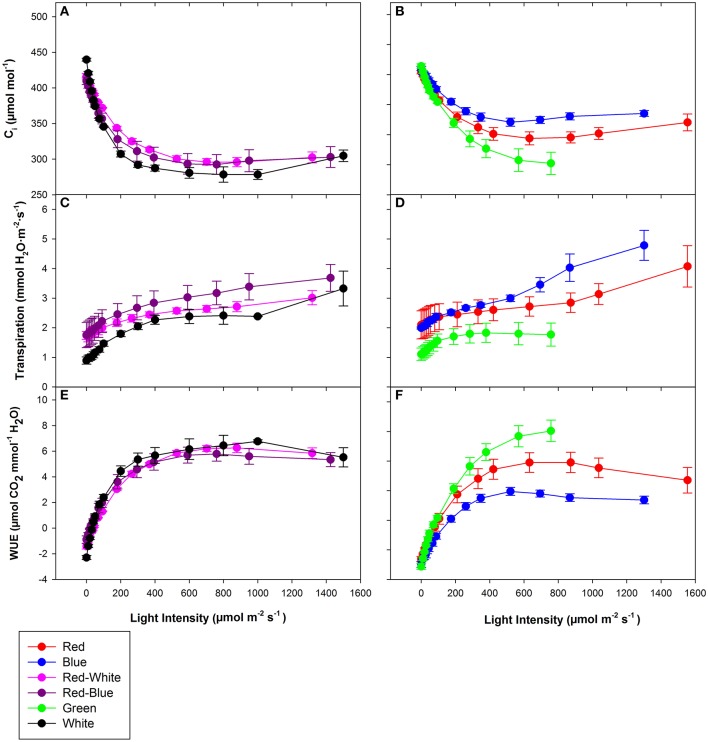
Major physiological traits of a tomato leaf eradiated with LEDs of different spectral quality. Leaf C_*i*_ **(A,B)**, leaf transpiration rate **(C,D)**, and leaf WUE **(E,F)** of tomato grown in growth chambers under fluorescent light. The data represent data points that each are the mean of 3 different leaves of 3 different plants ± their respective standard error at that light intensity.

The objective of the tomato leaf studies summarized in Figures 6, 7 as well as Table 1 were primarily to determine if the single major photosynthetic organ on the plant behaved in a manner similar to that of the whole plant canopy with respect to exposure with a wavelength specific LED fixture. Figures 6A, 7A,C,E show leaf responses to the spectra of selected commercially available combinations of LEDs. For ease of representation and visibility, Figures 6B, 7B,D,F show the effect of specific wavelength, monochromatic LED fixtures which highlights B, R, and G lights. Taken together, the commercially available combinations of LEDs produced very similar CO_2_ and H_2_O gas exchange (Figures 6A, 7A,C,E). However, when testing the monochromatic LEDs, the data showed clear differences in all leaf parameters when exposed to B, R, and G spectrum of different intensity (Figures 6B, 7B,D,F). Interestingly, even though CO_2_ gas exchange (Figure 6B) indicate that the B and G LEDs produced similar NCER values when compared to the R LED, calculations of C_*i*_ (Figure 7B), transpiration rates (Figure 7D), and WUE (Figure 7F) were very different under the B and G LEDs.

**Table 1:** A summary of the major physiological traits determined by analysis of leaf gas exchanges shown in Figures 6, 7 above.

**Light Treatment**	**Light compensation point (μmol m^−2^ s^−1^)**	**Quantum efficiency**	**Pn**_**max**_			
			**NCER (μmol m^−2^ s^−1^)**	**C_*i*_ (μmol mol^−1^)**	**E (mmol H_2_O m^−2^ s^−1^)**	**WUE (μmol CO_2_/mmol H_2_O)**
Red-Blue	21.40 (1.06)^b^	0.0035 (8.8 × 10^−5^)^a^	20.75 (0.71)^a^	292.52 (13.80)^ab^	3.17 (0.40)^ab^	5.78 (0.57)^ab^
Red-White	40.55 (0.72)^a^	0.0034 (5.8 × 10^−5^)^a^	20.51 (0.28)^a^	296.04 (4.70)^ab^	2.63 (0.11)^ab^	6.20 (0.23)^ab^
White	32.22 (2.36)^ab^	0.0036 (3.3 × 10^−5^)^a^	18.47 (0.58)^ab^	278.39 (10.74)^ab^	2.41 (0.29)^ab^	6.44 (0.79)^ab^
Red	18.93 (0.18)^b^	0.0035 (1.2 × 10^−4^)^a^	18.80 (0.13)^ab^	295.44 (9.34)^ab^	2.84 (0.33)^ab^	5.90 (0.68)^ab^
Blue	37.98 (2.47)^a^	0.0029 (2.0 × 10^−4^)^a^	17.49 (0.05)^ab^	324.43 (5.86)^a^	3.45 (0.24)^a^	3.79 (0.23)^b^
Green	22.63 (2.15)^ab^	0.0039 (5.0 × 10^−4^)^a^	16.19 (1.98)^b^	252.23 (18.58)^b^	1.76 (0.39)^b^	8.04 (0.72)^a^

The light compensation point, quantum efficiency, and Pn_max_ are calculated by f = y_o_ + a(1−e^(−b*x)^) while C_i_, E, and WUE are values at 700 μmol m^−2^ s^−1^ PAR for each LED light treatment tested. All values represent 3 leaf replicates and with the standard error (±), shown in parentheses. Letters (a,b) indicate significant difference within a column as determined by a one-way ANOVA with a Tukey Kramer adjustment (p < 0.05).

The differences shown in Figures 6, 7 are summarized in Table 1. The light compensation points of commercial fixtures indicated a significant difference between the RB and RW LED treatment (Figure 6A′; Table 1). The light compensation point of B and R LED light treatments also produced a difference when comparing the monochromatic LED treatments (Figure 6B′; Table 1). However, both the B and RW treatments produced higher light compensation point than did the R and RB LED treatments (Table 1). Interestingly, when quantum efficiencies under each of the LEDs were calculated, no difference for the tomato leaves could be observed (Figures 6A,B; Table 1).

When comparing the leaf NCER and WUE of the blended LED mixtures, no differences were noted at any light intensity (Figures 6A,E; Table 1). Figure 6B appears to show a slight difference in NCER rates between the light curves of R compared with B and G LED lights. However, when Pn_max_ values were statistically analyzed at *p* < 0.05, no difference among any of the monochromatic LED lights was determined (Table 1). However, the RW and RB blended LEDs produced higher Pn_max_ than did the G monochromatic LED treatment (Table 1). The WUE of the G and B LEDs was markedly different (Figure 7F; Table 1). The differences in WUE seemed to be due more to parameters which reflect stomatal functions (Figures 7B,D).

## Discussion

### Water-use-efficiency of a leaf under wavelength specific LEDs

Decades of research using filtered white light sources and, more recently, LEDs have determined the photosynthetic response of single leaves of several plants to wavelength specific lighting (McCree, 1972; Goins et al., 1997; Sun et al., 1998; Hogewoning et al., 2010; Liu et al., 2011a). However, most studies have used plants which are grown under, or have had long-term acclimatization to the light of interest (Liu et al., 2012; Rabara et al., 2017). Doing so, the possibility that the plants have different morphological characteristics, such as leaf area, stomatal density, and leaf thickness is a major concern (Brazaityte et al., 2010; Liu et al., 2011b, 2012). Thus, if different physical attributes are present at the time measurements are taken, a comparison is not being made between similar leaves/plants. Therefore, the measurements may be a function of long-term exposure to wavelength specific lighting and not a short-term plant response.

Data presented in Figures 6, 7 represents leaves which have been grown under white light and placed under short-term, wavelength specific illumination. This allows for the comparison of similar leaves and how they respond to short-term exposure. The results presented show similar data to those aforementioned studies, indicating that plant responses to wavelength specific lighting is able to happen rapidly (Goins et al., 1997; Hogewoning et al., 2010; Liu et al., 2011a). In all studies it was determined that leaves exposed to a combination of RB light induced the highest photosynthetic response which is what is presented in Figure 6A and Table 1 (Goins et al., 1997; Matsuda et al., 2004; Hogewoning et al., 2010; Liu et al., 2011a). It is also of no surprise that the RW LED treatment produced a high Pn_max_ due to the amounts of both R and B light it is comprised of (Liu et al., 2011a).

The G light treatment produced the lowest Pn_max_ among all the light treatments (Table 1). However, at lower light intensities than Pn_max_, G light was seen to provide a higher photosynthetic rate than did B light (Figure 6B). These findings are in line with results observed in spinach, cabbage, corn, and *Miscanthus* × *giganteus* leaves (Sun et al., 1998, 2012). As expected, the observed photosynthetic responses are highly correlated with the action spectrum of leaves from various species (McCree, 1972). Of note, G light provided a slightly higher quantum efficiency than did the B light treatment (Table 1). Although this data is calculated using the incident light, it is similar to previous work done with spinach and cabbage leaves (Sun et al., 1998). While B light is known to be highly absorbed by chlorophyll *a* and *b*, it is also highly absorbed by flavonoids and carotenoids which either do not transfer energy to the reaction center or do so poorly (Sun et al., 1998). Thus, the absorption of B light by other molecules can account for the lower quantum efficiency which was observed (Sun et al., 1998; Table 1).

Photosynthetic rates are not the only parameter effected by wavelength specific lighting. During long-term acclimation to wavelength specific light, stomatal morphology, density, and opening rates have been known to change (Liu et al., 2011a,b; Wang et al., 2016). Current studies analyzing stomatal function are mostly done using leaves which have been under long-term acclimation to wavelength specific light (Liu et al., 2011a, 2012). Internal CO_2_ concentration (C_i_), transpiration rates, and WUE values presented in Figure 7 indicated the immediate effect wavelength specific lighting on non-acclimated leaves. A rapid response of stomata when exposed to wavelength specific lighting has also been seen in rice leaves (Qu et al., 2016). At a light level of 700 μmol m^−2^ s^−1^, B light produced higher transpiration rates and C_i_ values than G light, and subsequently lower WUE (Figure 7; Table 1). Stomatal opening of *Xanthium strumarium* L., tomato, corn, and *M. giganteus* leaves show similar responses in stomatal function between B and G light (Sharkey and Raschke, 1981; Liu et al., 2012; Sun et al., 2012).

The underlying biochemical mechanism of which B and G light control stomatal functioning has been greatly studied. Blue light has been shown to cause hyperpolarization of guard cells leading to an increase in ion influx, causing stomatal opening (Assmann et al., 1985; Kinoshita and Shimazaki, 1999; Kinoshita et al., 2001; Shimazaki et al., 2007; Zhao et al., 2012). Green light, however, has been shown to have an antagonistic effect on stomatal opening in a variety of plant species (Zeiger and Zhu, 1998; Frechilla et al., 2000; Talbott et al., 2002). The interplay between B and G light effects on stomatal functioning are explanations for the differences in WUE, C_i_, and transpiration rates seen in Figure 7 and Table 1.

### Whole plant, diurnal patterns of biomass accumulation

Taken together, both the lisianthus and tomato whole plant diurnal patterns of CO_2_ and H_2_O exchanges showed that the two LED systems produce significantly lower daily WUE than did the traditional HPS (Figures 2, 3, 4). Although differences in transpiration and WUE were observed, the LEDs and the HPS are comparable in terms of supporting similar biomass gain and the daily carbon budgets (Figures 2, 3).

Plants which were grown under supplemental light produced little or no difference on a whole plant NCER basis, and only lisianthus plants grown under HPS light produced a higher photosynthetic rate (Figure 4B). However, an increase in end biomass production was seen from all of the supplemental light conditions (Figure 5). An increase in biomass production due to the use of supplemental lights has also been seen in cucumber and tomato greenhouse production in various studies (Hao and Papadopoulos, 1999; Dream et al., 2014). Results displayed in Figure 5 for both tomato and lisianthus crops indicate no difference between the different supplemental light treatments used here which is confirmed by the results for tomatoes presented by Dream et al. (2014) and Gomez and Mitchell (2015).

Although no increase was seen, previous literature has shown significant difference between some spectral qualities (Brazaityte et al., 2010; Liu et al., 2012). However, these studies used either a higher light intensity, sole source lighting, or a combination of both (Brazaityte et al., 2010; Liu et al., 2012). Thus, it is likely that at a higher light intensity that plant responses to the different spectral qualities were magnified and an increase in biomass production was able to be seen (Brazaityte et al., 2010; Liu et al., 2012). Also, lights used in these studies provided different spectrums than did the lights which were used in this study (Figure 1; Brazaityte et al., 2010; Liu et al., 2012).

### Whole plant diurnal patterns of NCER, transpiration, and WUE

Diurnal whole plant NCER showed little to no difference based on spectral quality in tomato or lisianthus (Figures 2, 3, 4). However, differences in both diurnal and daily averages were seen in both transpiration rate and WUE to some extent in both species (Figures 2, 3, 4). Thus, illuminating plants with different spectral quality is able to effect transpiration and WUE without major changes to whole plant NCER. These results seem to indicate that spectral quality can have an influence on stomata and their function without affecting the primary photosynthetic machinery. However, it is possible that plants which were grown under supplemental light have altered whole plant and leaf morphologies which has been well documented by others (Gay and Hurd, 1975; Goins et al., 1997; Liu et al., 2011b, 2012; Rabara et al., 2017).

Differences were observed between non-acclimated and acclimated lisianthus and tomato plants in Figures 2, 3, respectively. While it is shown in Figure 5, that these plants have different physical sizes, it is also likely that mutual shading is affecting light interception within the plant. Thus, like the different plant morphologies which are possible between plants growing under different spectra of lights, it is also possible that these differences are due to the inherent difference between plants grown with or without supplemental lighting (Gay and Hurd, 1975; Kumar et al., 2016).

Liu et al. (2011b) have provided evidence indicating the anatomical differences of tomato leaf stomata incurred by light of different spectral qualities. Light treatments which provide high amounts of B light, showed not only increased stomatal aperture but also an increased number of stomata when plants were grown under a G, O, or dysprosium lamp control (Liu et al., 2011b). Thus, it is possible increased transpiration and decreased WUE from the two LED lights were due to anatomical differences which has been induced by the B component of the lights.

Like tomato leaf studies, the two LED lights which were used in this experiment both contained higher amounts of R and B light than did the HPS light source (Figure 1A). Thus, it is also possible that stomatal opening is due strictly to the spectral quality they were analyzed under (Iino et al., 1985; Assmann, 1993; Kinoshita et al., 2001). Further, the spectrum of an HPS light is stronger in the G light region of the visible spectrum than either RB LED, which may further verify studies done by Frechilla et al. (2000) and Talbott et al. (2002).

Of note is the diurnal pattern shown in both transpiration rate and WUE for both species analyzed (Figures 2, 3). In all cases, the stomata which are responsible in part for controlling both of those parameters, seem to follow a defined circadian rhythm under all light treatments only with a translation upwards (transpiration rates) or downwards (WUE) from the LED lights. Plants, like other biological organisms, express circadian rhythms which are able to exist in the absence of light and dark periods (McClung, 2006). Many processes within plants follow a circadian rhythm which include germination, stomatal movement, gas exchange, gene expression and general growth (Cumming and Wagner, 1968; McClung and Kay, 1994).

Within *Arabidopsis* seedlings, cryptochrome (CRY) proteins show a diurnal pattern reaching maximum expression level a few hours after a light was turned on and subsequently declining there after (Toth et al., 2001). The use of *Arabidposis cry1cry2* double mutants displayed a reduced stomatal opening in response to B light which indicates an important role of CRY in stomatal opening (Mao et al., 2005). The diurnal pattern of CRY shown by Toth et al. (2001) follows closely with the transpiration rates shown in Figures 2, 3 and in Dodd et al. (2004) and thus may be a function of CRY involvement in stomatal opening (Mao et al., 2005).

Cryptochrome is a known B light receptor within both plants and animals (Chaves et al., 2011; Christie et al., 2015). Interestingly, the increase in transpiration rate and subsequent decrease in WUE are seen under both the RB and RW LED treatments which contain a higher component of B light than the HPS light (Figure 1A; Table 1; Supplementary Table 1). With CRY and other B light photoreceptors having a large influence on stomatal opening, the parallel can be drawn that the increase in transpiration rate coupled with the decrease of WUE under the RB and RW treatments is a function of the increased B light component acting on stomata.

The increase in transpiration rate during the night period which is seen in lisianthus and, to a dampened effect, in tomatoes provides evidence that stomatal functioning is not only monitored by light. Similar results have been reported by Dodd et al. (2004) indicating a rise in transpiration rate during the end of the night period anticipating dawn. Stomatal oscillation patterns presented in Dodd et al. (2004) and Gorton et al. (1989) under continuous light conditions indicate an engrained stomatal circadian rhythm which is not controlled solely by environmental conditions such as light.

During the winter months in greenhouses, not only is light a limiting factor, but humidity is generally lower. This has implications with the way stomata function and causes stomata to be in a more closed state (Lange et al., 1971). Causing stomatal closure will decrease the transpiration rate of the whole plant which can cause imbalances in vital micronutrient uptake which are important in various physiological process (Srivastava and Gupta, 1996; Xu et al., 2000; Baligar et al., 2001; Rouphael and Colla, 2005; Heckman, 2007; Alloway, 2008). The utilization of the RB and RW LEDs can help with the uptake of important nutrients during times of stress due to their ability to control stomatal functioning and increase transpiration rates and thus increase nutrient uptake allowing for proper plant growth.

In summary, understanding the effects of wavelength specific LED lighting on both a leaf and whole plant basis are important. Diurnal patterns of whole plant gas exchanges of CO_2_ and H_2_O for both tomato and lisianthus indicated clearly an increase in transpiration rates under both RB and RW LED lights compared to HPS lighting and similar photosynthetic and respiratory rates. Although, no differences were seen in NCER or end biomass between those lights, RB and RW LEDs produced statistically lower WUE than did the HPS light. Examination of monochromatic LED lights at the leaf level showed that adjusting B, R, and G wavelengths altered CO_2_ and H_2_O exchanges that accounted for the differences in the WUE patterns that were detected with the large commercially available luminaries. Taken together, both leaf and diurnal whole plant data show the importance in supplemental light selection for greenhouse production. In commercial production of both valuable vegetable and cut flower crops, the wavelength of artificial lighting may heavily influences WUE and subsequent nutrient uptake. Little is known about the relationship between WUE, nutrient balance, and spectral quality. The lack of data linking WUE, nutrient balance, and spectral quality have largely been ignored with respect to physiology, breeding studies, and phenology of potential new lines for commercial production.

## Author contributions

All authors listed, have made substantial, direct and intellectual contribution to the work, and approved it for publication.

### Conflict of interest statement

The authors declare that the research was conducted in the absence of any commercial or financial relationships that could be construed as a potential conflict of interest.

## Supplementary material

Supplementary Figure 1 Whole plant net carbon exchange system allowing for non-destructive biomass estimation as well as calculation of whole plant photosynthetic, respiration, and transpiration rates. The new modified system employs six, rather than four, polycarbonate/glass chambers similar to those described previously (Dutton et al., 1988; Leonardos et al., 2003). Light was provided from either a HPS, RB LED, or RW LED from Philips Lighting Company and LSGC, respectively. Lights were rotated among the chambers between runs to negate any chamber effects. Each chamber had its own temperature, CO_2_, humidity, and light sensors allowing for individual control of said chamber. Each chamber is 32″ × 18″ × 18″ with a total volume of 200 L. The system had two modes of use, an “open” and “closed” mode. In both modes, compressed air generated by the University of Guelph is scrubbed free of CO_2_ via a purge gas generator (CO_2_ Adsorber, Puregas, Broomfield, CO, USA). CO_2_ was then added back into the system at 400 μL L^−1^. Mixed gas was then pumped to the system via stainless steel piping and series of solenoid valves (ASCO RedHAT II, Florham Park, NJ, USA). CO_2_ and water measurements were checked every 20 s in sequential chambers (1 to 6) by infrared gas analyzer (IRGA; Li-COR CO_2_/H_2_O gas analyzer 840, Lincoln, NE, USA) in the “open” mode, allowing for adjustment of all chambers. In the “closed” mode, both the inlet and outlet solenoid valves of one chamber were closed allowing for the determination of the depletion of CO_2_ within said chamber. Sampling took place for 90 s with the first 30 s being excluded from calculations via Equation 1.1 where Vol is the chamber volume (L); *C*_*i*_ is the initial CO_2_ concentration during NCER measurement (μL L^−1^); *C*_*f*_ is the final CO_2_ concentration (μL L^−1^); 0.0821 is the gas constant (L°K^−1^ mol^−1^); T is the temperature (°K) and Δt is the elapse time during sampling (s). After 90s, the chamber was returned to the “open” mode and the next chamber was then set to the “closed” mode and sampled. One chamber was sample at a time with the other five chambers in the open mode. Sample was done in sequential chambers throughout the entirety of an experiment. $NCER=\frac{vol\left(c_{i}-c_{f}\right)}{0.0821*T*\Delta t}$.

Supplementary Table 1 Percentage of blue, green, yellow, orange, and red wavelengths within 714 light spectra provided in Figure 1.
