# Supplementary figures and images for: The Effect of Spectral Quality on Daily Patterns of Gas Exchange, Biomass Gain, and Water-Use-Efficiency in Tomatoes and Lisianthus: An Assessment of Whole Plant Measurements
Source: Front Plant Sci. 2017 Jun 20;8:1076. doi: 10.3389/fpls.2017.01076 (PMC5477295; doi:10.3389/fpls.2017.01076)

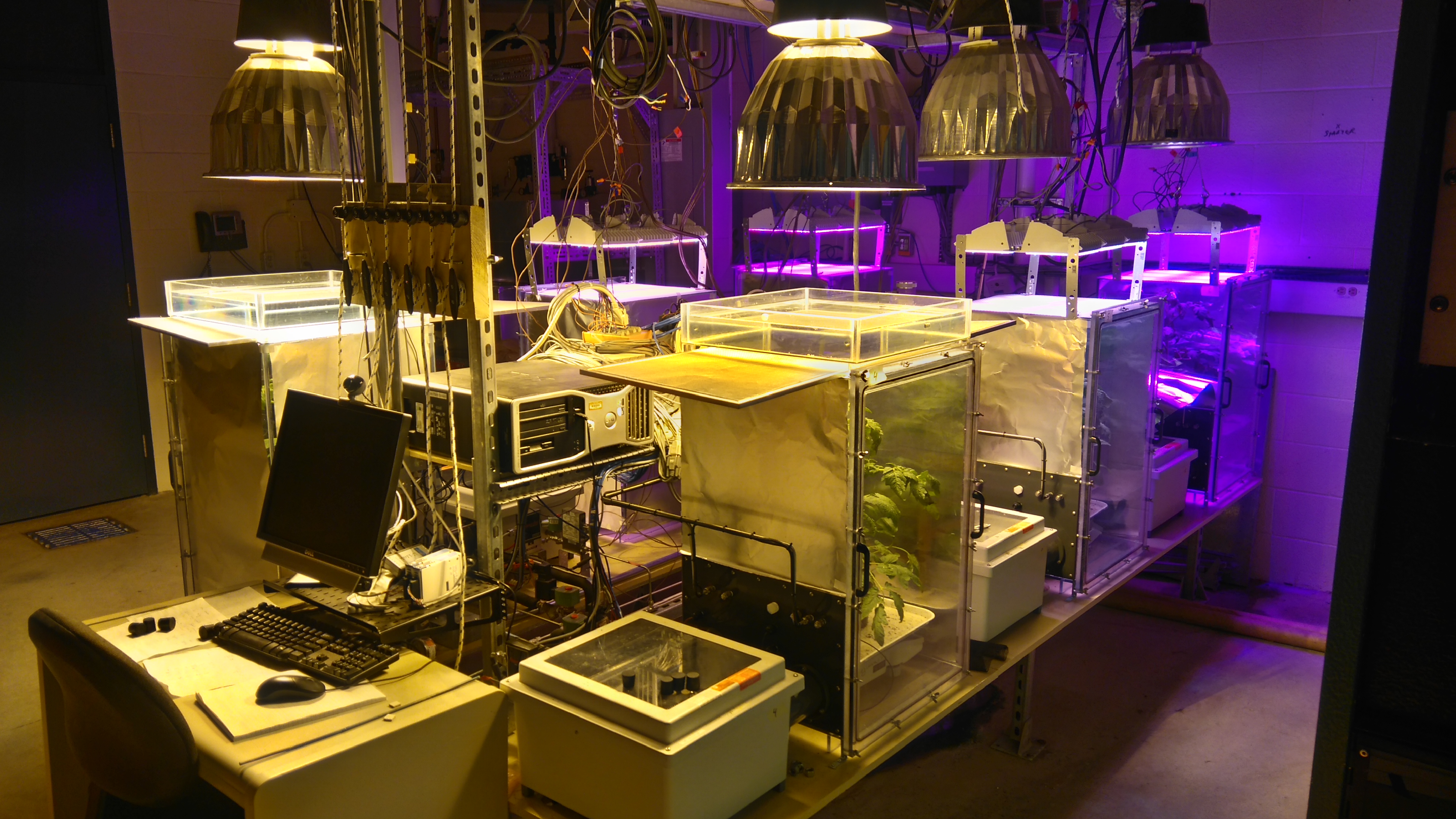

Supplement: Supplementary Figure 1 — Whole plant net carbon exchange system allowing for non-destructive biomass estimation as well as calculation of whole plant photosynthetic, respiration, and transpiration rates. The new modified system employs six, rather than four, polycarbonate/glass chambers similar to those described previously (Dutton et al., 1988; Leonardos et al., 2003). Light was provided from either a HPS, RB LED, or RW LED from Philips Lighting Company and LSGC, respectively. Lights were rotated among the chambers between runs to negate any chamber effects. Each chamber had its own temperature, CO2, humidity, and light sensors allowing for individual control of said chamber. Each chamber is 32″ × 18″ × 18″ with a total volume of 200 L. The system had two modes of use, an “open” and “closed” mode. In both modes, compressed air generated by the University of Guelph is scrubbed free of CO2 via a purge gas generator (CO2 Adsorber, Puregas, Broomfield, CO, USA). CO2 was then added back into the system at 400 μL L−1. Mixed gas was then pumped to the system via stainless steel piping and series of solenoid valves (ASCO RedHAT II, Florham Park, NJ, USA). CO2 and water measurements were checked every 20 s in sequential chambers (1 to 6) by infrared gas analyzer (IRGA; Li-COR CO2/H2O gas analyzer 840, Lincoln, NE, USA) in the “open” mode, allowing for adjustment of all chambers. In the “closed” mode, both the inlet and outlet solenoid valves of one chamber were closed allowing for the determination of the depletion of CO2 within said chamber. Sampling took place for 90 s with the first 30 s being excluded from calculations via Equation 1.1 where Vol is the chamber volume (L); Ci is the initial CO2 concentration during NCER measurement (μL L−1); Cf is the final CO2 concentration (μL L−1); 0.0821 is the gas constant (L°K−1 mol−1); T is the temperature (°K) and Δt is the elapse time during sampling (s). After 90s, the chamber was returned to the “open” mode and the next chamber was then set to the “cl [file Image1.JPEG]
