# Supplementary material for: The Effect of Spectral Quality on Daily Patterns of Gas Exchange, Biomass Gain, and Water-Use-Efficiency in Tomatoes and Lisianthus: An Assessment of Whole Plant Measurements
Source: Front Plant Sci. 2017 Jun 20;8:1076. doi: 10.3389/fpls.2017.01076 (PMC5477295; doi:10.3389/fpls.2017.01076)
Supplement: Supplementary Table 1 — Percentage of blue, green, yellow, orange, and red wavelengths within 714 light spectra provided in Figure 1. [file Table1.docx]

|  | **Wavelength composition (%)** | **Blue (400-495nm)** | **Green (495-570nm)** | **Yellow (570-590nm)** | **Orange (590-620nm)** | **Red (620-750nm)** |
| --- | --- | --- | --- | --- | --- | --- |
| **Growth lights** | Fluorescent | 15.78 | 41.44 | 8.49 | 24.22 | 10.13 |
|  | HPS | 2.09 | 20.98 | 30.31 | 33.89 | 13.81 |
|  | Red-Blue | 17.78 | 0.55 | 0.18 | 1.40 | 80.10 |
|  | Red-White | 10.36 | 21.12 | 3.19 | 3.62 | 61.67 |
| **PAR 38 floodlights** | White | 8.85 | 36.27 | 13.48 | 20.80 | 21.48 |
|  | Red-Blue | 27.96 | 0.31 | 0.09 | 0.97 | 70.67 |
|  | Red-White | 11.68 | 22.61 | 3.46 | 3.77 | 58.75 |
|  | Red | 0.36 | 0.35 | 0.14 | 1.54 | 97.62 |
|  | Blue | 98.26 | 1.06 | 0.09 | 0.13 | 0.47 |
|  | Green | 2.03 | 94.14 | 2.69 | 0.85 | 0.61 |
